# Hyperammonemia after capecitabine associated with occult impairment of the urea cycle

**DOI:** 10.1002/cam4.2036

**Published:** 2019-04-11

**Authors:** Gilbert Chu, Julia Salzman

**Affiliations:** ^1^ Department of Medicine Stanford University Stanford California; ^2^ Department of Biochemistry Stanford University Stanford California

## Abstract

**Background:**

Cancer patients receiving chemotherapy often complain of “chemobrain” or cognitive impairment, but mechanisms remain elusive.

**Methods:**

A patient with gastric cancer developed delirium and hyperammonemia after chemotherapy with the 5‐fluorouracil pro‐drug capecitabine. Exome sequencing facilitated a search for mutations among 43 genes associated with hyperammonemia and affecting the urea cycle directly or indirectly.

**Results:**

The patient's urea cycle was impaired by capecitabine‐induced liver steatosis, and portosystemic shunting of gut ammonia into the systemic circulation. The patient was also heterozygous for amino acid substitution mutations previously reported to create dysfunctional proteins in 2 genes, ORNT2 (ornithine transporter‐2 for the urea cycle), and ETFA (electron transport flavoprotein alpha for fatty acid oxidation). The mutations explained the patient's abnormal plasma amino acid profile and exaggerated response to allopurinol challenge. Global population variations among the 43 hyperammonemia genes were assessed for inactivating mutations, and for amino acid substitutions predicted to be deleterious by complementary algorithms, SIFT and PolyPhen‐2. One or 2 deleterious mutations occur among the 43 genes in 13.9% and 1% of individuals, respectively.

**Conclusions:**

Capecitabine and 5‐fluorouracil inhibit pyrimidine biosynthesis, decreasing ammonia utilization. These drugs can induce hyperammonemia in susceptible individuals. The risk factors of hyperammonemia, gene mutations and liver dysfunction, are not rare. Diagnosis will trigger appropriate treatment and ameliorate brain toxicity.

## 1 INTRODUCTION

Cancer patients often complain of “chemobrain,” but without an understanding of the mechanism, physicians have limited options to ameliorate the symptoms. Neuropsychological deficits occur in 32% of breast cancer patients receiving high dose chemotherapy.^1^ These deficits are associated with abnormal brain white matter organization in magnetic resonance imaging.^2^ However, mechanisms for cognitive impairment have remained elusive.^3^

Here, we report a patient with delirium and hyperammonemia after treatment with capecitabine, a pro‐drug of 5‐fluorouracil (5‐FU). Genetic analysis and imaging revealed that the patient had several risk factors for hyperammonemia. We propose a mechanism for how 5‐FU or capecitabine triggers hyperammonemia in the setting of these risk factors. Of note, 5‐FU and capecitabine are among the most commonly used anti‐cancer drugs, with roles in cancers of the head and neck, esophagus, stomach, pancreas, colon, and breast. Furthermore, prompt diagnosis of hyperammonemia can lead to reversal of cognitive impairment.

## 2 CASE REPORT

A 67 year‐old female with gastric adenocarcinoma underwent subtotal gastrectomy and Roux‐en‐Y gastrojejunostomy. She then received adjuvant treatment consisting of 2 cycles of adjuvant carboplatin and capecitabine (1000 mg/m^2^ twice daily for 14 days), followed by radiation therapy to the tumor bed with concurrent capecitabine (1000 mg/m^2^ twice daily). Each course of treatment was free of mucositis, diarrhea, or hand‐foot syndrome, but was associated with extreme lethargy. On the third cycle of carboplatin and capecitabine, she self‐administered folate 1 mg/d. On days 5 to 14 of capecitabine, she suffered frank delirium. She presented to a local emergency room on day 6 where computerized tomography (CT) scan of the brain was normal, and was sent home without a diagnosis. Seven days after finishing capecitabine, her oncologist noted persistent confusion and gait ataxia, and admitted her to the hospital (hereafter referred to as “first hospitalization”). Plasma ammonia was 158 µmol/L (normal less than 30 µmol/L). With oral lactulose, plasma ammonia declined to 29 µmol/L, confusion resolved, and lactulose was discontinued.

Seven months after first hospitalization, the patient was hospitalized for hyperammonemia associated with a urinary tract infection, and was restarted on daily lactulose. Nineteen and 21 months after the first hospitalization, the patient was hospitalized with hyperammonemia. Lactulose was started, and supplemented successively with neomycin, rifaximin, and glycerol phenyl butyrate.

The patient suffered weight loss from 69 to 42 kg and progressive muscle weakness. Six years after first hospitalization, sensory and nerve conduction studies and needle electromyography were normal. Laminectomy at L4‐L5 failed to relieve leg weakness.

Eight years after first hospitalization, the patient was hospitalized for hyperammonemia 3 times in 2 months. CT scan detected a portosystemic from the inferior mesenteric vein to the internal iliac vein, which in retrospect, had been present immediately after gastrectomy (Figure 3). Liver biopsy showed minimal (5%) macrovesicular steatosis, mild periportal fibrosis, and mild to moderate parenchymal iron deposition, which occurs in the setting of a portosystemic shunt.^4^ The shunt was occluded via percutaneous transhepatic catheter. Plasma ammonia declined to normal, demonstrating the physiological importance of the shunt. The patient was able to discontinue neomycin, and glycerol phenylbutyrate, but continues taking rifaxamin, and intermittent lactose in the form of Kristalose.

## 3 RESULTS

### 3.1 Capecitabine and 5‐FU can induce encephalopathy when the urea cycle is impaired

**Figure 1:**
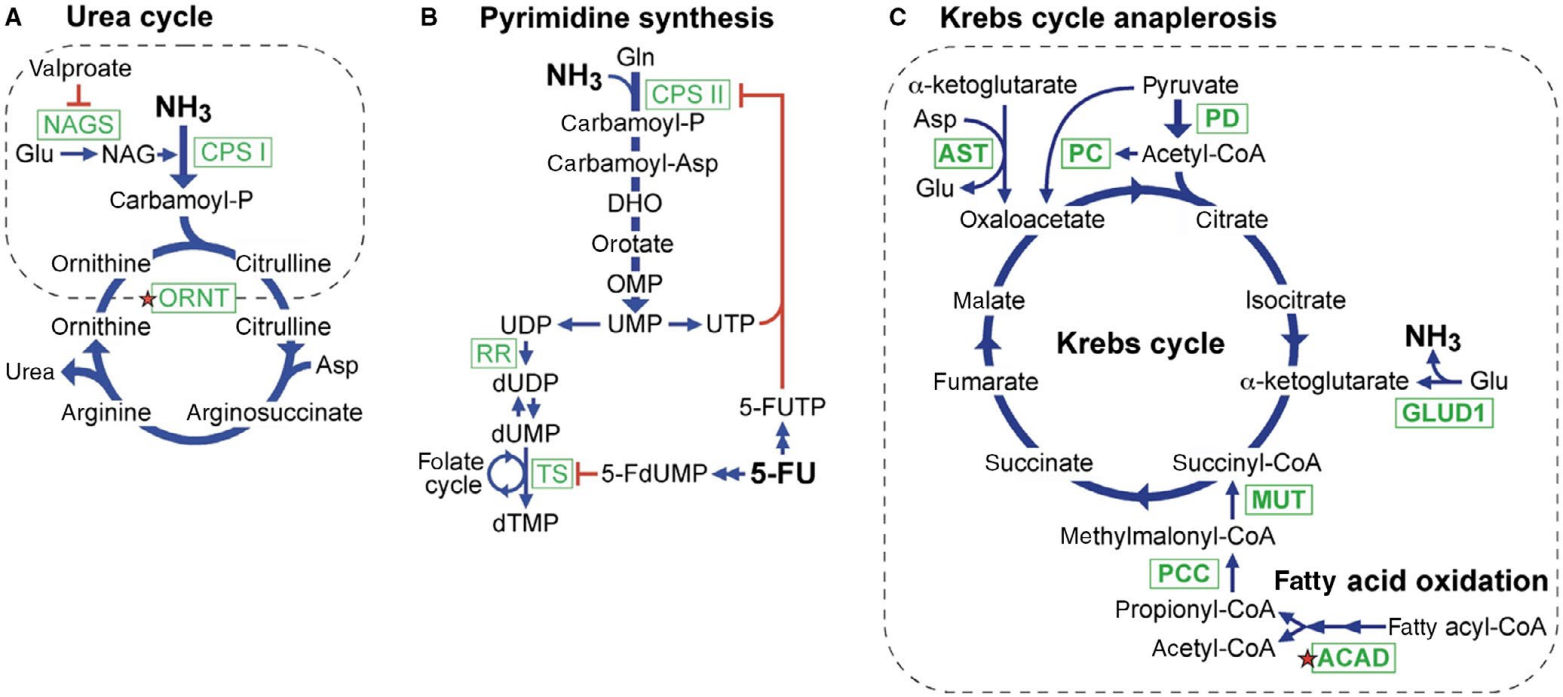
Ammonia metabolism pathways. A, Urea cycle. B, Pyrimidine biosynthesis. C, Krebs cycle anaplerosis pathways. Green boxes mark key enzymes, dotted lines encircle steps inside mitochondria, and red stars mark defects in the patient. Additional abbreviations: DHO, dihydroorotate; MUT, methylmalonyl‐CoA mutase; NAG, N‐acetylglutamate; NAGS, N‐acetylglutamate synthase; OMP, orotidine monophosphate; PCC, propionyl‐CoA carboxylase; RR, ribonucleotide reductase; TS, thymidylate synthase

Hyperammonemia occurs when the urea cycle is severely impaired, which can occur from gene mutations, damage to the liver (the major site for the urea cycle), or valproate (Figure 1A). Hyperammonemia also occurs when the urea cycle is mildly impaired, if accompanied by increased ammonia production, which can arise from gut flora, urinary tract infection with urea‐splitting bacteria, or a catabolic state from weight loss. We hypothesized that capecitabine and 5‐FU can induce hyperammonemia when the urea cycle is mildly impaired by blocking ammonia utilization.

The urea cycle eliminates ammonia by carbamoyl phosphate synthase type I (carbamoyl phosphate synthase I [CPS I]), which localizes to mitochondria and catalyzes the reaction:

$$\text{2ATP}+{\text{HCO}}_{\text{3}}^{-}+{\text{NH}}_{\text{4}}^{+}\to \text{2ADP}+\text{carbamoyl}\;\text{phosphate}+\text{Pi}.$$

Pyrimidine biosynthesis utilizes ammonia (Figure 1B) via carbamoyl phosphate synthase type II (CPS II), which localizes to the cytosol and catalyzes the reaction:

$$\text{glutamine}+{\text{CO}}_{\text{2}}+\text{2 ATP}+{\text{H}}_{\text{2}}\text{O}\to \text{carbamoyl}\;\text{phosphate}+\text{glutamate}+\text{2 ADP}+\text{Pi.}$$

Ammonia is the molecular substrate for CPS II, which has a Km for ammonia comparable to CPS I.^5^

The end product of pyrimidine biosynthesis, uridine‐5'‐triphosphate (UTP) inhibits CPS II by negative feedback.^6^ The 5‐FU metabolite 5‐FUTP inhibits CPS II in yeast,^7^ and presumably in humans. We propose that 5‐FU interferes with ammonia utilization by inhibiting CPS II, thus increasing the ammonia load to the urea cycle.

**Figure 2:**
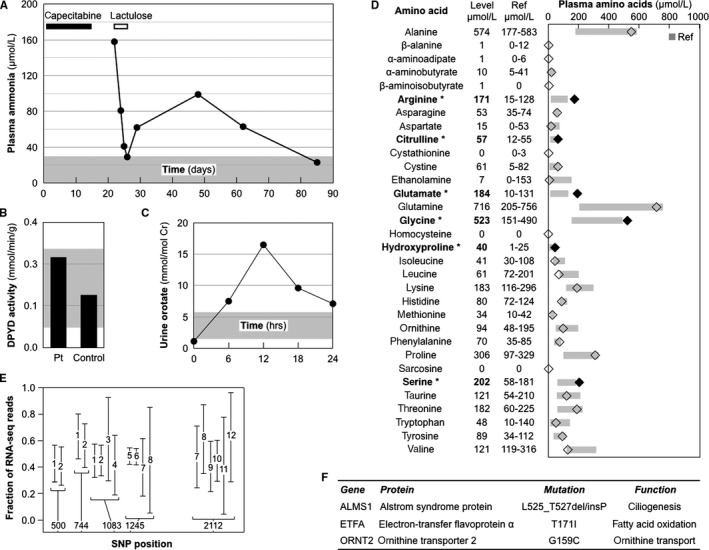
Biochemical and genetic analysis of the patient. Normal ranges in panels A, B, C, and D appear in gray. A, Delayed plasma ammonia clearance. The patient received capecitabine on days 1‐14 (black bar), and lactulose on days 21‐23 (white bar). B, Normal dihydropyrimidine dehydrogenase (DYPD) activity in the patient (Pt). C, Excessive urine orotate after allopurinol challenge. D, Plasma amino acids. Levels were measured on 6 different days when plasma ammonia levels ranged from 19 to 180 µmol/L (average, 69 µmol/L). (Abnormal amino acid levels in bold and black diamonds). E, No effect of SLC7A7 splice donor polymorphism (position 1083) on its RNA levels. RNA sequencing data are shown from 12 acute myelogenous leukemias (1‐12) heterozygous for SNPs in at least one of 5 positions. F, Relevant genes with deleterious mutations

The patient had no previous history of hyperammonemia. Nevertheless, after discharge from first hospitalization, ammonia levels rose before returning to normal over the course of 2 months (Figure 2A). This indicated grossly slowed elimination of ammonia due to occult impairment of the urea cycle, and we embarked on a biochemical and genetic investigation.

### 3.2 The patient had complex defects affecting the urea cycle

To determine the cause of hyperammonemia, we performed biochemical and genetic analyses, after obtaining consent according to a protocol approved by the Stanford Administrative Panel on Human Subjects. Dihydropyrimidine dehydrogenase (DPYD) deficiency, which interferes with 5‐FU catabolism, has been associated with 5‐FU‐induced neurotoxicity manifesting as gait ataxia, confusion, stupor, and even coma.^8^, ^9^ However, DPYD enzymatic activity was normal (Figure 2B), and the common mutations, DPYD*2A (IVS14 +1 G>A) and DPYD*13 (1679T>G; I560S), were absent.

Laboratory tests suggested a defect in the urea cycle. Challenge with 300 mg of allopurinol detects defects in ornithine transcarbamylase or mitochondrial ornithine transport. Such defects increase carbomoyl phosphate levels. Urinary orotate increases due to allopurinol blockade of OMP decarboxylase‐catalyzed conversion of OMP to UMP in the pyrimidine synthesis pathway (Figure 1B). Baseline urine orotate was 1.0 nmol/mol creatinine, and increased to a peak level of 16.5 nmol/mol creatinine after allopurinol (Figure 2C), far greater than the normal peak level of 4.6 ± 2.8 nmol/mol creatinine for adult women.^10^ However, a single urea cycle defect failed to explain the spectrum plasma amino acid elevations (Figure 2D).

### 3.3 Hyperammonemia has been associated with 43 genes

We identified a total of 43 genes associated with hyperammonemia (Table S1). Online Mendelian Inheritance in Man (OMIM) yielded 41 of these 43 genes after using the search word “hyperammonemia” and eliminating false hits. Two additional genes, ORNT2 and ORNT3, encode mitochondrial membrane transporters that act in parallel with the classical urea cycle ornithine transporter ORNT1.^11^, ^12^ DPYD does not appear in Table S1 because DPYD deficiency has not been associated with 5‐FU‐induced hyperammonemia, despite its association with 5‐FU‐induced neurotoxicity.

Hyperammonemia genes participate in the urea cycle, the Krebs cycle, mitochondrial fatty acid oxidation, and branched chain amino acid degradation. In searching for a unified mechanism for hyperammonemia among these genes, we noted that the nonurea cycle genes facilitate anaplerosis,^13^ which replenishes Krebs cycle intermediates and competes with the urea cycle for glutamate and aspartate (Figure 1A,C). For example, GLUD1 mutations cause autosomal dominant hyperinsulinism‐hyperammonemia syndrome by generating hyperactive GLUD1, which generates ammonia by glutamate deamination,^14^ and competes with the urea cycle for glutamate. Pyruvate carboxylase and pyruvate dehydrogenase mutations both disrupt conversion of pyruvate to oxaloacetate. To maintain Krebs cycle oxaloacetate, aspartate transaminase (AST) activity increases, thus competing with the urea cycle for aspartate.^15^

Fatty acid oxidation gene mutations in proprionic acidemia or methylmalonic acidemia deplete Krebs cycle succinyl‐CoA. To maintain Krebs cycle succinyl‐CoA, GLUD1 activity increases,^16^ thus generating ammonia. Propionic and methylmalonic acidemias suppress the urea cycle by additional mechanisms. Injection of rats with propionic or methylmalonic acid leads to depletion of N‐acetylglutamate^17^; accumulation of propionyl‐CoA, which competitively inhibits N‐acetylglutamate synthase^18^; and accumulation of methylmalonyl‐CoA, which competitively inhibits pyruvate carboxylase.^19^

TUFM mutations reduce translation of mitochondrial proteins to cause a combined oxidative phosphorylation deficiency that disrupts fatty acid oxidation.^20^ HLCS, HMGCL, IVD, MCCC1, and MCCC2 mutations lead to accumulation of branched‐chain amino acids, which inhibit pyruvate dehydrogenase.^21^ In conclusion, mutations in the nonurea cycle genes share the common feature of affecting Krebs cycle anaplerosis, either competing with the urea cycle for glutamate and aspartate, or in the case of GLUD1, generating ammonia directly.

### 3.4 The patient carried deleterious mutations in 2 hyperammonemia genes

We analyzed the patient's exome in 2 stages. Stage 1 focused on the 43 hyperammonemia genes plus the DPYD gene. We failed to detect nonsense, invariant splice site, or insertion/deletion mutations, but did find 15 nonsynonymous single nucleotide polymorphisms (SNPs). Two SNPs were identified as deleterious mutations by 2 complementary algorithms: SIFT (Sorting Tolerant From Intolerant), which is based on evolutionary conservation^22^; and PolyPhen‐2 (Polymorphism Phenotyping version 2), which is based on sequence and structure‐based algorithms (Table S2).^23^

One mutation was in ETFA, which encodes the alpha subunit of ETF, an electron‐transfer‐flavoprotein linking acyl‐CoA dehydrogenase (ACAD) to the respiratory chain in the fatty acid oxidation pathway (Figure 1C). Mutations in ETFA cause glutaric acidemia IIA, 1 form of multiple acyl‐CoA dehydrogenase deficiency (Table S1). Simple heterozygous mutations in ETFA may be significant, since such mutations in ACAD cause adult onset disease.^24^ The patient's ETFA mutation encodes a T171I substitution, which decreases thermal stability of the protein, and is over‐represented in patients with the hyperammonemia‐associated disease of very‐long‐chain acyl‐CoA dehydrogenase deficiency.^25^

The second mutation was in ORNT2, which encodes a urea cycle ornithine transporter with 88% amino acid identity to ORNT1 (Figure 1A).^12^ Functional redundancy from ORNT2 may explain the mild phenotype of ORNT1 deficiency relative to other urea cycle disorders. The ORNT2 mutation encodes a G159C substitution, which decreases ornithine transport when expressed in ORNT1‐deficient cells.^12^ Defective ornithine transport was consistent with the patient's abnormal allopurinol challenge test (Figure 2C).

Among splice site SNPs, the strongest candidate was a homozygous SNP in SLC7A7 in the nonconsensus position SD‐2 of the splice donor consensus sequence, (A/C)**A**G|GUPuAGU>(A/C)**G**G|GUPuAGU. The allele frequency for this SNP in the general population is 0.386, and RNA sequence analysis showed no effect on SLC7A7 mRNA expression (Figure 2E). We concluded that the splice site SNP was benign, and inferred that weaker candidate splice site SNPs were also benign.

Stage 2 of the analysis searched the whole exome for overtly deleterious mutations in genes with roles in the urea cycle or Krebs cycle anaplerosis. The whole exome contained nonsense mutations in 48 genes; invariant splice site mutations in 35 genes; and insertion/deletion mutations in 7 genes (Tables S3‐S5). Polymorphisms in ACSM2A and ALMS1 were potentially relevant.

ACSM2A encodes an acyl‐CoA synthetase,^26^ which forms a thioester with CoA to activate medium chain fatty acids for beta‐oxidation.^27^ ACSM2A was heterozygous for nonsense mutation R115X, which truncates 462 of the 577 amino acids in the protein. However, ACSM2A shares the same tissue expression and 97.6% sequence identity with ACSM2B. In addition, ACSM2A has not been associated with hyperammonemia and has an allele frequency of 0.18 in East Asians (https://www.ncbi.nlm.nih.gov/projects/SNP/snp_ref.cgi?searchType=adhoc_search&type=rs&rs=rs59261767). Thus, the R115X mutation by itself is unlikely to be a major contributor to hyperammonemia, but could enhance the effect of the deleterious ETFA mutation, because both genes participate in fatty acid oxidation.

ALMS1 is mutated in the autosomal recessive disease Alstrom Syndrome, and required for the normal function of primary cilia in multiple tissues, including the liver.^28^ The patient was heterozygous for the ALMS1 mutation L525_T527delinsP, which replaces L525‐E526‐T527 with a helix destabilizing proline. This mutation is not among the 79 reported Alstrom Syndrome mutations, most of which are private,^29^ and thus represents a new private mutation. In summary, the patient was heterozygous for deleterious mutations in 2 hyperammonemia genes: ORNT1 for ornithine transport, and ETFA for fatty acid oxidation (red stars in Figure 1A,C), and heterozygous for a deleterious mutation in ALMS1 affecting liver function (Figure 2F).

### 3.5 Fatty acid oxidation defect explains abnormal plasma amino acid levels in the patient

The patient's ETFA mutation partially disrupted fatty acid oxidation, consistent with multiple observations of abnormal plasma amino acid levels (Figure 2D). Decreased fatty acid oxidation limits the supply of succinyl‐CoA to the Krebs cycle (Figure 1C). Anaplerosis responds by supplying upstream alpha‐ketoglutarate via increased glutamate to promote GLUD1 activity, consistent with the patient's increased glutamate levels. Anaplerosis also responds by supplying downstream succinate via the prolyl hydroxylase reaction:

$$\text{proline}+\text{alpha}-\text{ketoglutarate}+{\text{O}}_{\text{2}}\to \text{hydroxyproline}+\text{succinate}+{\text{CO}}_{\text{2}}.$$

Increased proline promotes increased prolyl hydroxylase activity to generate succinate and hydroxyproline. Increased proline and hydroxyproline levels are associated with ETFA mutations.^30^ Indeed, the patient's levels were at the upper range of normal and mildly elevated, respectively.

Decreased fatty acid oxidation limits the supply of acetyl‐CoA to the Krebs cycle (Figure 1C). To conserve acetyl‐CoA while maintaining oxaloacetate for the Krebs cycle, anaplerosis increases the conversion of aspartate to oxaloacetate and glutamate by AST. This is consistent with the patient's low normal plasma aspartate. Decreased aspartate slows the conversion of citrulline to arginosuccinate in the urea cycle (Figure 1A), consistent with her mildly elevated plasma citrulline.

Decreased fatty acid oxidation limits the supply of short chain fatty acids, suppressing glycine decarboxylation,^31^ consistent with the patient's elevated plasma glycine. Glycine and serine undergo reversible interconversion by serine hydroxymethyltransferase, consistent with her elevated plasma serine. Decreased fatty acid oxidation decreases fatty acids that bind and activate PPAR gamma and delta,^32^ which are required for arginase induction.^33^ Decreased arginase inhibits the conversion of arginine to ornithine and urea (Figure 1A), consistent with the patient's elevated plasma arginine.

## 4 DISCUSSION

### 4.1 Several sources of urea cycle dysfunction contributed to hyperammonemia after capecitabine

The literature contains a single report of hyperammonemia and encephalopathy after treatment with the oral 5‐FU pro‐drug capecitabine.^34^ However, that report did not analyze the patient for a possible mechanism. Here we report a second case, and went further by studying our patient to discover several otherwise occult impairments of the urea cycle. We propose that such impairments place patients at risk for hyperammonemia after 5‐FU because its conversion to 5‐UTP decreases ammonia utilization by inhibiting pyrimidine biosynthesis.

The patient had several impairments of the urea cycle. The mutation in ornithine transporter (ORNT2) affected the urea cycle. Indeed, the increased urine orotate peak after allopurinol challenge supported a defect in the ornithine transport step of the urea cycle. The mutation in ETFA may have impacted fatty acid oxidation. Indeed, the abnormalities in the patient's plasma amino acids were consistent with a mild fatty acid oxidation defect.

Chemotherapy with 5‐FU produces fatty liver disease on CT scans in 47% of patients,^35^ and significantly abnormal liver function tests in 39.6% of patients compared to 16.1% of patients randomized to observation alone.^36^ The patient did indeed develop fatty liver disease as documented by serial CT scans. The patient's heterozygous mutation in ALMS1 may have been a contributing factor, since liver dysfunction is a component of autosomal recessive Alstrom Syndrome.

**Figure 3:**
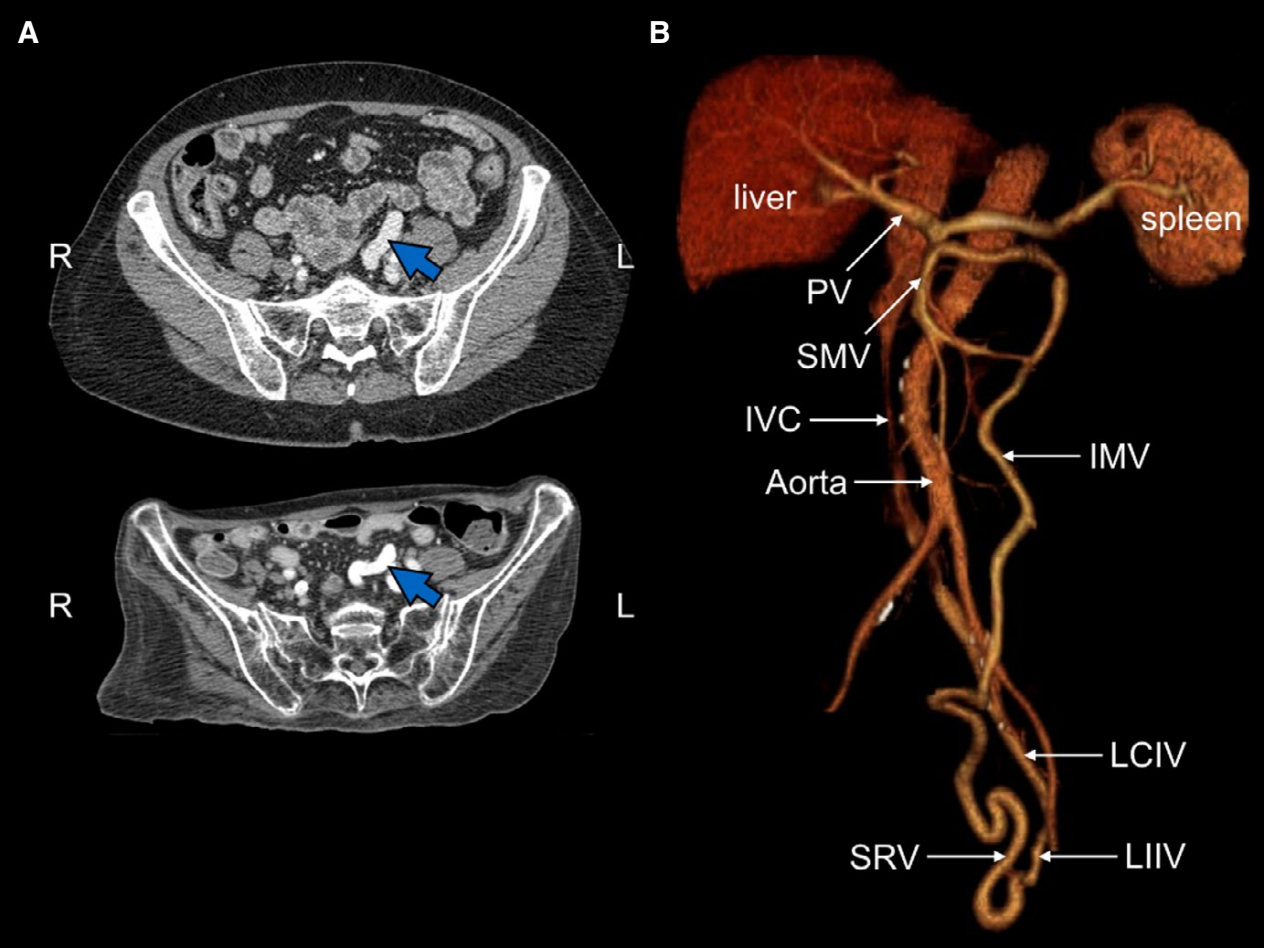
Imaging of portosystemic shunt. A, CT scans. Blue arrow indicates dilated superior rectal vein, present from 6 months prior to first hospital admission (upper panel) to 8 years later (lower panel). Note dramatic weight loss. B, CT reconstruction of vascular system. Note shunt of superior rectal vein (SRV) to the left internal iliac vein (LIIV). The shunt produces early opacification of the left common iliac vein (LCIV), but not the right common iliac vein. Other abbreviations: IVC, inferior vena cava; IMV, inferior mesenteric vein, PV, portal vein; SMV, superior mesenteric vein

The patient's portosystemic shunt compromised hepatic blood supply, which exacerbated liver dysfunction and redirected ammonia absorbed from the gut to the systemic circulation and away from the urea cycle in the liver. The shunt was documented in a series of CT scans from 2 months after partial gastrectomy to 8 years later (Figure 3). Portosystemic shunts associated with hyperammonemia have been reported for gastric cancers.^37^, ^38^, ^39^

By themselves, the shunt and deleterious mutations were not sufficient to cause hyperammonemia. Capecitabine provided the precipitating insult by inhibiting pyrimidine biosynthesis and thus compromising ammonia utilization. The patient's self‐administration of folate explains why encephalopathy was most severe on the last capecitabine cycle. Folate enhances 5‐FU inhibition of thymidylate synthase,^40^ thus increasing UTP levels. As a result, both 5‐FUTP and UTP contributed to inhibition of CPS II, the rate‐limiting step of pyrimidine biosynthesis (Figure 1B).

### 4.2 Hyperammonemia was treated successfully

After institution of a low protein diet, lactulose, neomycin, rifaximin, and glycerol phenylbutyrate the patient did well for 4 years before suffering another hospitalization for hyperammonemia. Subsequent episodes of hyperammonemia may have occurred in the setting of progressive changes in the liver due to compromised hepatic blood supply. At 8 years, liver biopsy performed during the shunt occlusion procedure showed periportal fibrosis and iron deposition, changes consistent with a chronic portosystemic shunt. The dramatic improvement in blood ammonia after shunt occlusion indicated that it was a contributing factor to the first hyperammonemia hospitalization.

The patient's leg weakness and associated weight loss (Figure 3A) were not due to common causes. Common neuromuscular defects were ruled out by normal nerve conduction and normal electromyogram studies. Neural impingement was ruled out by lack of benefit from laminectomy.

Multiple acyl‐CoA dehydrogenase deficiency due to mutation in ETFA can cause muscle weakness and weight loss. Late onset muscle weakness in adulthood occurs in multiple acyl‐CoA dehydrogenase deficiency. Most patients with late onset respond to pharmacological doses of riboflavin, likely because high concentrations of the riboflavin co‐factor induce proper folding of mutant acyl‐CoA dehydrogenase protein.^41^ Eight years after first hospitalization, the patient started riboflavin 100 mg daily, and noted an immediate improvement in appetite accompanied by weight gain. Unfortunately, she continues to suffer leg weakness, perhaps because of chronic deconditioning and advancing age.

### 4.3 Hyperammonemia may not be a rare complication of 5‐FU/capecitabine

“Idiopathic” hyperammonemia is currently believed to be an uncommon complication of chemotherapy, particularly with treatment‐induced tumor lysis of hematological malignancies.^42^ For solid tumors treated with an unusually high dose of 5‐FU (2600 mg/m^2^ in a 24‐hour infusion), 16 of 280 patients (5.7%) suffered hyperammonemia and encephalopathy.^43^ Such high 5‐FU doses within a 24‐hour time interval are now rarely administered, and the authors did not propose a mechanism for how 5‐FU would trigger hyperammonemia.

We propose that 5‐FU and its oral pro‐drug capecitabine can induce hyperammonemia by inhibiting pyrimidine biosynthesis. We further propose that hyperammonemia can occur in patients harboring otherwise occult defects in the urea cycle. Mutations in any of 43 genes confer risk for hyperammonemia. The magnitude of risk depends on the number of affected genes and severity of the mutations. Deleterious mutations in hyperammonemia genes are not rare. Sixteen of the 43 hyperammonemia genes have deleterious SNPs with allele frequencies of at least 0.0004 (Table S6). The sum of the allele frequencies for these 16 genes is 0.149. Thus, deleterious mutations occur in at least 1 gene in 13.9% of the population, and at least 2 genes in 1% of the global population (Table S7).

Several clinical risk factors confer risk for hyperammonemia by interfering with ammonia elimination (liver dysfunction, valproate, portosystemic shunts) or increasing ammonia production (urinary tract infection, tumor lysis, catabolic state from weight loss). The patient described here had multiple risk factors, but a single risk factor such as severe liver dysfunction could be sufficient. Patients treated 5‐FU or capecitabine may have multiple risk factors associated with their cancer, including 5‐FU‐induced liver damage, liver metastases, and weight loss.

The incidence of hyperammonemia after capecitabine may be more common than previously believed. Deleterious mutations and liver dysfunction are not uncommon in the general population. Liver dysfunction is common in cancer patients due to liver metastases or exposure to chemotherapy drugs such as capecitabine, 5‐FU, oxaliplatin, and irinotecan. Capecitabine is typically administered over 14‐day periods, potentially exposing patients to a prolonged period of hyperammonemia. Many patients may suffer cognitive dysfunction without the frank delirium in the patient reported here.

Finally, other pyrimidine and purine analogs employed as anti‐cancer agents, including gemcitabine, cytarabine, fludarabine, pentostatin and mercaptopurine, might also induce hyperammonemia. Awareness of risk factors and diagnosis of hyperammonemia will trigger effective treatments, preventing cognitive dysfunction, brain damage, and even death.

## CONFLICT OF INTEREST

None.
