# Supplementary material for: Hyperammonemia after capecitabine associated with occult impairment of the urea cycle
Source: Cancer Med. 2019 Apr 11;8(5):1996–2004. doi: 10.1002/cam4.2036 (PMC6536928; doi:10.1002/cam4.2036)
Supplement: Supplementary file 1 [file CAM4-8-1996-s001.pdf]

SUPPORTING INFORMATION

Table S1. Genes associated with hyperammonemia

Square brackets enclose protein function and disease, with the disease underlined.

Urea cycle genes

ALDH18A1	aldehyde dehydrogenase 18 member 1 [ornithine, arginine, proline biosynthesis, cutis laxa type IIIA]
ARG1	arginase, liver [argininemia]
ASS1	argininosuccinate synthase 1 [citrullinemia type I]
ASL	argininosuccinate lyase [argininosuccinic acidemia]
CPS1	carbamoyl phosphate synthase 1, mitochondrial
GLUL	glutamate-ammonia ligase [synthesis of glutamine from glutamate, congenital glutamine deficiency]
NAGS	N-acetylglutamate synthase
ORNT1	SLC25A15, solute carrier family 25 (mitochondrial carrier; ornithine transporter) member 15 [hyperornithinemia-hyperammonemia-homocitrullinuria (HHH) syndrome]
ORNT2	SLC25A2, solute carrier family 25 (mitochondrial carrier; ornithine transporter) member 2
ORNT3	SLC25A29, solute carrier family 25 (mitochondrial carnitine/acylcarnitine carrier protein CACL) member 29
OTC	ornithine transcarbamylase
SLC7A7	solute carrier family 7 (cationic amino acid transporter, y+ system) member 7 [Arg, Lys, ornithine transport in kidney and small intestine, lysinuric protein intolerance]
SLC25A13	solute carrier family 25 member 13 (citrin) [exchange of Asp for Glu across inner mitochondrial membrane, citrullinemia type II]

Fatty acid oxidation genes

ACADVL	acyl-CoA dehydrogenase, very long chain
ACADM	acyl-CoA dehydrogenase, C-4 to C-12 straight chain
CPT1A	carnitine palmitoyltransferase 1A (liver)
CPT2	carnitine palmitoyltransferase 2
ETFA	electron-transfer-flavoprotein, alpha polypeptide [glutaric acidemia IIA]
ETFB	electron-transfer-flavoprotein, beta polypeptide [glutaric acidemia IIB]
ETFDH	electron-transferring-flavoprotein dehydrogenase [glutaric acidemia IIC]
HADHA	hydroxyacyl-CoA dehydrogenase/3-ketoacyl-CoA thiolase/enoyl-CoA hydratase, alpha subunit
HADHB	hydroxyacyl-CoA dehydrogenase/3-ketoacyl-CoA thiolase/enoyl-CoA hydratase, beta subunit
HLCS	holocarboxylase synthase (biotin- (propionyl-CoA-carboxylase (ATP-hydrolysing)) ligase) [gluconeogenesis, branched chain amino acid catabolism]
HMGCL	3-hydroxymethyl-3-methylglutaryl-CoA lyase [final step in leucine degradation]
IVD	isovaleryl-CoA dehydrogenase [valine, leucine, isoleucine degradation, isovaleric acidemia]
LMBRD1	LMBR1 domain containing 1 [cobalamin transporter, homocystinuria-megaloblastic anemia type F]
MCCC1	methylcrotonoyl-CoA carboxylase 1 (alpha) [leucine catabolism]
MCCC2	methylcrotonoyl-CoA carboxylase 2 (beta) [leucine catabolism]
MLYCD	malonyl-CoA-decarboxylase [stimulates fatty acid oxidation by converting malonyl-CoA to acetyl-CoA, [combined malonic and methylmalonic acidemia]
MMAA	methylmalonic acidemia (cobalamin deficiency) cblA type [methylmalonic acidemia]
MMAB	methylmalonic acidemia (cobalamin deficiency) cblB type [methylmalonic acidemia]
MMACHC	methylmalonic acidemia (cobalamin deficiency) cblC type, with homocystinuria [methylmalonic acidemia]
MMADHC	methylmalonic acidemia (cobalamin deficiency) cblD type, with homocystinuria [methylmalonic acidemia]
MUT	methylmalonyl CoA mutase [isomerization of methylmalonyl-CoA to succinyl-CoA, methylmalonic acidemia]
PCCA	propionyl CoA carboxylase, alpha polypeptide [propionic acidemia]
PCCB	propionyl CoA carboxylase, beta polypeptide [propionic acidemia]
SLC25A20	solute carrier family 25 member 20 [carnitine/acylcarnitine translocase deficiency]
SLC22A5	solute carrier family 22 (organic cation/carnitine transporter) member 5 [carnitine deficiency]

Other anaplerosis genes

DLAT	dihydrolipoamide S-acetyltransferase [in mitochondrial complex that converts pyruvate to acetyl-CoA]
GLUD1	glutamate dehydrogenase 1 [hyperinsulinism-hyperammonemia syndrome]
PC	pyruvate carboxylase [mitochondrial pyruvate oxidation to oxaloacetate]
PDHA1	pyruvate dehydrogenase (lipoamide) alpha 1 [in mitochondrial complex that converts pyruvate to acetyl-CoA]
TUFM	Tu translation elongation factor, mitochondrial [protein translation in mitochondria, combined oxidative phosphorylation deficiency]

Table S2. Patient missense and splice site mutations
For the 43 hyperammonemia genes plus DPYD, we obtained allele frequencies and disease associations from the SNP database https://www.ncbi.nlm.nih.gov/snp. Abbreviations: NA, not applicable; NV, normal variant based on SIFT and PolyPhen2 predictions and high allele frequency; Ref DNA, reference DNA sequence; SD, splice donor site.

Gene	*SNP ID*	*Ref DNA*	*Patient DNA*	*Mutation*	*SIFT*	*PolyPhen2*	*Allele freq*	*Conclusion*
CPS1	rs1047891	C	C/A	T1406N	tolerated	benign	0.288	NV[A]
ORNT1	rs17849654	A	A/T	I254L	tolerated	benign	0.323	NV
ORNT2	rs3749780	G	G/A	V226I	tolerated	benign	0.128	NV
	rs10075302	G	G/T	G159C	damaging	possibly damaging	0.043	damaging[B]
MMAB	rs9593	T	T/A	M239K	tolerated	benign	0.487	NV
LMBRD1	rs12648	A	A/T	D469E	tolerated	benign	0.385	NV
HLCS	rs61732502	G	G/A	V96I	tolerated	benign	0.058	NV
CPT2	rs1799821	G	G/A	V368I	tolerated	benign	0.413	NV
	rs776754218	C	C/T	P520L	tolerated	benign	0.00005	benign[C]
MUT	rs8589	A	A/G	I671V	tolerated	benign	0.451	NV
	rs2229385	G	G/A	A499T	tolerated	benign	0.086	benign[D]
ETFA	rs1801591	C	C/T	T171I	damaging	damaging	0.051	damaging[E]
ETFDH	rs11559290	C	C/T	T31I	tolerated	benign	0.327	NV
SLC7A7	rs8018462	A	G/G	SD-2	NA	NA	0.418	benign[F]
DPYD	rs1801159	A	A/G	I543V	tolerated	benign	0.185	NV[G]
	rs1801265	T	C/C	C29R	tolerated	benign	0.260	NV[G]

Notes
A T1406N has no effect on plasma arginine concentrations[1].
B G159C shows decreased activity in cells transfected with a cDNA expression vector[2].
C P520L is predicted to preserve protein function and is not among the 64 mutations found in neonatal or severe infantile carnitine palmitoyltransferase II deficiency[3].
D A499T confers normal enzymatic activity[4].
E T171I affects thermal stability of the ETF enzyme and is over-represented among patients with very-long-chain acyl-CoA dehydrogenase deficiency[5].
F The splice donor SD-2 polymorphism had no effect on RNA, as determined by RNA-sequence analysis of 12 acute myelogenous leukemias (Fig. 2E).
G Patient had normal enzymatic activity (Fig. 2B).

Table S3. Patient nonsense mutations

A nonsense mutation was deemed homozygous if we observed multiple homozygous reads at both the mutation site and adjacent SNPs (in parentheses). Other nonsense mutations were heterozygous.

Gene	*Mutation*	*Homozygous reads*	*Protein*	*Function*
PDE4DIP	p.R785X		phosphodiesterase 4D interacting protein	anchors cAMP PDE4D to Golgi
CPN2	p.Q509X	12 (61)	carboxypeptidase N subunit 2	carboxypeptidase
SLC6A18	p.Y319X		solute carrier family 6 member 18	Na-dependent neutral a.a. transporter, role in iminoglycinuria & hyperglycinuria
ZNF117	p.R428X		zinc finger protein 117	possible transcriptional regulator
FSCN3	p.R423X		fascin-3	actin bundling protein
LPL	p.S474X		lipoprotein lipase precursor	triglyceride hydrolysis
VPS13B	p.Y413X		vacuolar protein sorting-assoc protein 13B	vesicle-mediated transport
IFNE	p.Q71X		interferon epsilon precursor	interferon
CTBP2	p.Q445X		C-terminal-binding protein 2	corepressor of transcriptional regulators
OR1B1	p.R192X	20 (145)	olfactory receptor 1B1	olfactory receptor
OR10X1	p.W66X	47 (118)	olfactory receptor 10X1	olfactory receptor
OR51Q1	p.R236X		olfactory receptor 51Q1	olfactory receptor
OR4X2	p.Y27X		olfactory receptor 4X2	olfactory receptor
OR4X1	p.Y273X	108 (71)	olfactory receptor 4X1	olfactory receptor
OR4C3	p.W174X		olfactory receptor 4C3	olfactory receptor
OR4A15	p.Q256X		olfactory receptor 4A15	olfactory receptor
OR4C16	p.Q17X		olfactory receptor 4C16	olfactory receptor
OR5AR1	p.Q19X		olfactory receptor 5AR1	olfactory receptor
OR10V1	p.Q123X		olfactory receptor 10V1	olfactory receptor
SLC22A10	p.W96X		solute carrier family 22 member 10	organic anion transporter
KLRK1	p.W74X		NKG2-D type II integral membrane protein	receptor on NK cells
HEBP1	p.Q147X		heme-binding protein 1	removes free porphyrinogens
ACSM2A	p.R115X		acyl-coA synthetase medium chain member 2A	medium chain fatty acid:CoA ligase
TM4SF5	p.S160X		transmembrane 4 L6 family member 5	tetraspannin protein, cell proliferation
MAP2K3	p.Q73X		dual specificity MAP kinase kinase 3	component of the MAP kinase cascade
HAP1	p.W104X		huntingtin-assoc protein 1 isoform 3	associates with huntingtin
CDC27	p.R631X		cell division cycle protein 27 homolog	anaphase promoting complex component
SERPINB7	p.R266X		serpin peptidase inhib, clade B member 7	inhibits lysine-specific proteases
RHPN2	p.Q378X		rhophilin-2	organization of actin cytoskeleton
USP29	p.Y913X	129	ubiquitin carboxyl-terminal hydrolase 29	de-ubiquitinating (DUB) enzyme
WFDC8	p.R5X		WAP four-disulfide core dom protein 8	protease inhibitor
UBE2NL	p.L89X		putative ubiq-conjug enzyme E2 N-like	91% identical to UBE2N, DNA repair
NEIL1	p.R325X		endonuclease VIII-like 1	DNA glycoslylase in BER
ZACN	p.Q281X	55 (101)	Zn-activated ligand-gated ion channel	?
ZC3H3	p.S880X	6 (21)	Zn finger CCCH dom-containing protein 3	?
MAGEE2	p.E120X	33 (106)	melanoma-associated antigen E2	?
MAGEB16	p.R272X		melanoma-associated antigen B16	?
C5orf49	p.R72X		hypothetical protein LOC134121	?
UNC93A	p.W151X		protein unc-93 homolog A isoform 2	?
CC2D2B	p.Q320X		protein CC2D2B isoform 2	?
PRAMEF2	p.E211X		PRAME family member 2	?
MOBKL2C	p.R24X		mps1 binder kin. activator-like 2C isoform	?
SPATA8	p.R34X		spermatogenesis-associated protein 8	?
TPTE	p.R211X		putative Tyr-protein phosphatase TPTE	?
C1orf227	p.R37X		hypothetical protein LOC149643	?
VWA3B	p.E219X		von Willebrand factor A domain-containing	?
SERHL2	p.Q212X		serine hydrolase-like protein 2	?
FTHL17	p.E148X		ferritin heavy polypeptide-like 17	?

Table S4. Patient invariant splice site mutations
The table shows genes with mutations in the splice donor site (SD) invariant GT, or splice acceptor site (SA) invariant AG. An invariant splice site mutation was deemed homozygous if we observed multiple homozygous reads at both the mutation site and adjacent SNPs (in parentheses). Other invariant splice site mutations were heterozygous.

Gene	*Mutation*	*Homozygous reads*	*Protein*
TCTEX1D1	SD1		tctex1 domain-containing protein 1
HTR3D	SD1	65 (65)	5-hydroxytryptamine receptor 3D isoform 3
WDR67	SD1		WD repeat-containing protein 67 isoform 1
C9orf43	SD1		hypothetical protein LOC257169
NUCB2	SD1	74	nucleobindin-2 precursor
GSTT2	SD1		glutathione S-transferase theta-2
SAA1	SD2	3 (115)	serum amyloid A protein preproprotein
AGL	SA-2		glycogen debranching enzyme isoform 1
ZFP91	SA-2		zinc finger protein 91 homolog isoform 2
GREB1	SA-1		protein GREB1 isoform c
MUC7	SA-1		mucin-7 precursor
XRCC4	SA-1	14 (24)	DNA repair protein XRCC4 isoform 2
OAS1	SA-1	43 (86)	2'-5'-oligoadenylate synthase 1 isoform 1
C13orf26	SA-1		hypothetical protein LOC122046
C17orf57	SA-1		EF-hand dom-containing protein C17orf57
LILRA2	SA-1		leukocyte Ig-like rec subfamily

Table S5. Patient insertion/deletion mutations
The table shows insertions or deletions (indels) sequenced more than once. Indels for CLCA4, SMARCA2, and ATN1 occur in repeated amino acid sequences, and are therefore presumed to be polymorphisms.

Gene	*Mutation*	*Reads*	*Protein*
CLCA4	2 a.a. del: (PT)5 > (PT)4	2	Calcium-activated chloride channel regulator 4 precursor
OR7C2	frameshift	2	Olfactory receptor 7C2
C21orf6	frameshift	3	Uncharacterized protein C21orf62
GPATCH4	frameshift	3	G patch domain-containing protein 4
SMARCA2	1 a.a. del: (Q)23 > (Q)22	4	Probable global transcription activator SNF2L2
ATN1	5 a.a. del: (Q)19 > (Q)14	2	Atrophin-1 (Dentatorubral-pallidoluysian atrophy)
ALMS1	L525_T527del/insP	2	Alstrom syndrome protein 1

Table S6. Population deleterious SNPs among 43 hyperammonemia genes

We obtained data from the SNP database https://www.ncbi.nlm.nih.gov/snp. For each gene, the table shows the SNP with the highest allele frequency of the SNPs deemed pathogenic by the 1000 Genomes phase 3 genotype data from 2500 worldwide individuals, as released in the May 2013 dataset. The table is restricted to the 16 genes with pathogenic SNPs with global mean allele frequencies of at least 0.0004, which corresponds to 2 observations in 1088 individuals (or 2176 chromosomes).

Gene	*Chromosome*	*SNP id*	*Protein change*	*Allele frequency*
ACADM	1	rs147559466	E43K	0.0020
ACADVL	17	rs28934585	P65L	0.0391
ASL	22	rs28941471	R190Q	0.0010
ASS1	9	rs35269064	R108L	0.0050
CPS1	2	rs200214298	G169R	0.0004
CPT1A	11	rs2229738	A275T	0.0238
CPT2	1	rs1871748	S565C	0.0016
IVD	15	rs28940889	A284V	0.0004
MCCC2	5	rs119103219	E99Q	0.0004
		rs150591260	V339M	0.0004
MMAB	12	rs35648932	A135T	0.0030
MMACHC	1	rs398124292	frameshift	0.0010
MUT	6	rs547709692	T297I	0.0022
ORNT1	13	rs141028076	intron variant	0.0010
OTC	X	rs1800328	Q270R	0.0167
PDHA1	X	rs2229137	M282L	0.0495
SLC22A5	5	rs11568514	Y473D	0.0016

Table S7. Population frequency of pathogenic SNPs

The sum of the allele frequencies for the 16 genes in Table S6 was 0.149. Five additional genes had pathogenic SNPs with global mean allele frequencies of 0.0002, providing a total contribution of 0.001. The remaining 21 genes did not have pathogenic SNPs observed in the 1000 Genomes phase 3 genotype data. Thus, the average number of deleterious SNPs in the global population was estimated to be 0.150. This number was used as the parameter λ for the Poisson distribution, which estimates the probability for n pathogenic SNPs, $P(n) = (\lambda^n/n!) \exp(-\lambda)$. Shown are the estimated fraction of the population carrying: zero, P(0); one or more, P(≥1); and two or more, P(≥2) deleterious SNPs.

Sum of allele frequencies	*P(0)*	*P(≥1)*	*P(≥2)*
0.150	0.861	0.139	0.010

Materials and Methods

DPYD analysis

DPYD enzymatic activity was measured in peripheral blood lymphocytes from the patient and an age-matched healthy control. Samples were harvested at the same time, and shipped on dry ice. The laboratory of Dr. Robert Diasio (Mayo Clinic, Rochester, MN) measured DPYD enzymatic activity and performed DPYD mutation analysis.

Exome sequencing

After obtaining informed consent from the Stanford University Administrative Panel for the Protection of Human Subjects, the patient's exome was analyzed using biotinylated RNA baits to capture exome DNA[6], and then sequenced to 84-fold coverage, with a range of 6 to 498 reads (Hudson-Alpha Institute, Huntsville, AL). To determine if amino acid substitutions affected protein function, we applied two algorithms. SIFT (Sorting Tolerant From Intolerant) assumes that important positions in the amino acid sequence of a protein have been conserved during evolution, and predicts the effects of substitutions at each position in the amino acid sequence[7]. PolyPhen-2 (Polymorphism Phenotyping version 2) uses sequence-based and structure-based algorithms to predict the functional importance of an amino acid substitution[8]. Allele frequencies and other information for specific genes were obtained from GeneCards (http://www.genecards.org/).

RNA sequence analysis

To determine whether the SD-2 splice donor site SNP (A/C)**A**G|GUPuAGU > (A/C)**G**G|GUPuAGU at position 1083 affects RNA levels, we analyzed published RNAseq data from 12 acute myelogenous leukemias heterozygous for SNPs in at least one of five positions (Fig. 2E): SRR061899, SRR061823, SRR061886, SRR061900, SRR061757, SRR054844, SRR061824, SRR061898, SRR061897, SRR061758, SRR061885, SRR054845, respectively (http://0-www.ncbi.nlm.nih.gov.elis.tmu.edu.tw/Traces/sra/sra.cgi?view=search_obj).

Measurement of plasma ammonia levels

To measure plasma ammonia levels, Stanford Health Care Clinical Laboratory placed the blood sample on ice, and analyzed the sample within 15 minutes. Sample handling is important, because samples not placed on ice, or analyzed after a longer delay yield falsely elevated plasma ammonia levels[9, 10].

Supporting Information References

1. Moonen RM, Reyes I, Cavallaro G, Gonzalez-Luis G, Bakker JA, Villamor E. The T1405N carbamoyl phosphate synthetase polymorphism does not affect plasma arginine concentrations in preterm infants. PLoS One. 2010;5:e10792.
2. Camacho JA, Rioseco-Camacho N, Andrade D, Porter J, Kong J. Cloning and characterization of human ORNT2: a second mitochondrial ornithine transporter that can rescue a defective ORNT1 in patients with the hyperornithinemia-hyperammonemia-homocitrullinuria syndrome, a urea cycle disorder. Mol Genet Metab. 2003;79:257-271.
3. Isackson PJ, Bennett MJ, Lichter-Konecki U, et al. CPT2 gene mutations resulting in lethal neonatal or severe infantile carnitine palmitoyltransferase II deficiency. Mol Genet Metab. 2008;94:422-427.
4. Champattanachai V, Ketudat Cairns JR, Shotelersuk V, et al. Novel mutations in a Thai patient with methylmalonic acidemia. Mol Genet Metab. 2003;79:300-302.
5. Bross P, Pedersen P, Winter V, et al. A polymorphic variant in the human electron transfer flavoprotein alpha-chain (alpha-T171) displays decreased thermal stability and is overrepresented in very-long-chain acyl-CoA dehydrogenase-deficient patients with mild childhood presentation. Mol Genet Metab. 1999;67:138-147.

6. Gnirke A, Melnikov A, Maguire J, et al. Solution hybrid selection with ultra-long oligonucleotides for massively parallel targeted sequencing. Nat Biotechnol. 2009;27:182-189.
7. Kumar P, Henikoff S, Ng PC. Predicting the effects of coding non-synonymous variants on protein function using the SIFT algorithm. Nat Protoc. 2009;4:1073-1081.
8. Adzhubei IA, Schmidt S, Peshkin L, et al. A method and server for predicting damaging missense mutations. Nature Methods. 2010;7:248-249.
9. Howanitz JH, Howanitz PJ, Skrodzki CA, Iwanski JA. Influences of specimen processing and storage conditions on results for plasma ammonia. Clinical Chemistry. 1984;30:906-908.
10. Maranda B, Cousineau J, Allard P, Lambert M. False positives in plasma ammonia measurement and their clinical impact in a pediatric population. Clin Biochem. 2007;40:531-535.
